# The Effect of Statins on Mortality in Septic Patients: A Meta-Analysis of Randomized Controlled Trials

**DOI:** 10.1371/journal.pone.0082775

**Published:** 2013-12-31

**Authors:** Laura Pasin, Giovanni Landoni, Maria Lourdes Castro, Luca Cabrini, Alessandro Belletti, Paolo Feltracco, Gabriele Finco, Andrea Carozzo, Roberto Chiesa, Alberto Zangrillo

**Affiliations:** 1 Department of Anesthesia and Intensive Care, San Raffaele Scientific Institute, Milan, Italy; 2 Maieutics Foundation, Milan, Italy; 3 Anesthesiology Department, Centro Hospitalar Lisboa Central, Lisbon, Portugal; 4 Department of Anesthesia and Intensive Care, University Hospital of Padua, Padua, Italy; 5 Department of Medical Sciences “M. Aresu”, Cagliari University, Cagliari, Italy; 6 Department of Vascular Surgery, San Raffaele Scientific Institute, Milan, Italy; University of Sao Paulo Medical School, Brazil

## Abstract

**Objective:**

Statins are among the most prescribed drugs worldwide and their recently discovered anti-inflammatory effect seems to have an important role in inhibiting proinflammatory cytokine production, chemokines expression and counteracting the harmful effects of sepsis on the coagulation system. We decided to perform a meta-analysis of all randomized controlled trials ever published on statin therapy in septic patients to evaluate their effect on survival and length of hospital stay.

**Data sources and study selection:**

Articles were assessed by four trained investigators, with divergences resolved by consensus. BioMedCentral, PubMed, Embase and the Cochrane Central Register of clinical trials were searched for pertinent studies. Inclusion criteria were random allocation to treatment and comparison of statins versus any comparator in septic patients.

**Data extraction and synthesis:**

Data from 650 patients in 5 randomized controlled studies were analyzed. No difference in mortality between patients receiving statins versus control (44/322 [14%] in the statins group vs 50/328 [15%] in the control arm, RR = 0.90 [95% CI 0.65 to 1.26], p = 0.6) was observed. No differences in hospital stay (p = 0.7) were found.

**Conclusions:**

Published data show that statin therapy has no effect on mortality in the overall population of adult septic patients. Scientific evidence on statins role in septic patients is still limited and larger randomized trials should be performed on this topic.

## Introduction

Discovered by Akira Endo in 1970s [1] and taken by more than 20 millions of Americans, 3-hydroxy-3-methylglutaryl coenzyme A (HMG-CoA) reductase inhibitors (statins) are, nowadays, the most prescribed drugs in the world.

They are widely used in medical practice as cholesterol-lowering agents and their beneficial effects on vascular diseases, reducing the risk of myocardial infarction and prolonging life, have been demonstrated in several clinical trials [2], [3], even if statin therapy does not eliminate cardiovascular risk [4], [5]. In the last few years a beneficial effect of statins on the outcome of other severe disease such as cancer and infections [6], [7] has been hypothesized. This “pleiotropic” effect seems to be related to their potential modulation of both innate and adaptative immune system and anti-inflammatory effects [8]–[10]. By inhibiting tissue factor expression and reducing prothrombin fragment levels [11] and by strongly increasing the expression of thrombomodulin [12], statins seem to have an important role in counteracting the harmful effects of sepsis on the coagulation system. Moreover numerous studies suggest inhibitory effects of statins on proinflammatory cytokine production (Interferon-γ, tumor necrosis factor-α, interleukin (IL-1β and IL-6) and on chemokines (chemokines CCL2, CCL7, CCL13, CCL18, CXCL1) expression [13]–[15]. Accordingly, many observational studies suggested that statin treatment may be associated with a better prognosis in severe bacterial infections.

Since new randomized trials have recently appeared on this topic [16]–[18] we decided to perform a meta-analysis of all randomized controlled trials ever performed on statin therapy in septic patients to evaluate its effect on survival and length of hospital stay.

## Methods

### Search Strategy

Pertinent studies were independently searched in BioMedCentral, PubMed, Embase, and the Cochrane Central Register of clinical trials (updated September 1^st^ 2013) by four trained investigators. The full PubMed search strategy aimed to include any randomized study ever performed in humans with statins in sepsis or infectious diseases and is presented in the supplemental material. In addition, backward snowballing was employed (i.e., scanning of references of retrieved articles and pertinent reviews) and international experts were contacted for further studies. No language restriction was imposed.

### Study Selection

References obtained from database and literature searches were first independently examined at a title/abstract level by four investigators, with divergences resolved by consensus, and then, if potentially pertinent, retrieved as complete articles. The following inclusion criteria were used for potentially relevant studies: random allocation to treatment (statins versus any comparator with no restrictions on dose or time of administration) and studies involving septic patients. The exclusion criteria were: duplicate publications either acknowledged or not (in this case we referred to the first article published while retrieved data from the article with the longest follow-up available), non-adult patients and lack of data on main outcomes. Compliance to selection criteria and selected studies for the final analysis were independently assessed by two investigators, with divergences finally resolved by consensus. Primary outcome was mortality at the longest follow-up available in each single study. Secondary outcome was hospital length of stay (HLOS).

### Internal Validity and Risk of Bias Assessment

The internal validity and risk of bias of included trials was appraised by two independent reviewers according to Cochrane Collaboration methods [19], with divergences resolved by consensus. Publication bias was assessed by visually inspecting funnel plots (Figures S1 and S2).

### Data Analysis and Synthesis

Computations were performed with Review Manager version 5.2. Hypothesis of statistical heterogeneity was tested by means of Cochran Q test, with statistical significance set at the two-tailed 0.10 level, whereas extent of statistical consistency was measured with I^2^, defined as 100% X (Q-df)/Q, where Q is Cochran's heterogeneity statistic and df the degrees of freedom. Binary outcomes were analysed to compute the individual and pooled risk ratio (RR) with pertinent 95% confidence interval (CI), by means of the same models as just described. Binary outcomes from individual studies were analysed to compute individual and pooled risk ratio (RR) with pertinent 95% confidence interval (CI), by means of inverse variance method and with a random-effect model (which better accommodates clinical and statistical variations). Mean differences (MD) and 95% confidence intervals were computed for continuous variables using the same models as just described. Sensitivity analyses were performed by sequentially removing each study and reanalysing the remaining dataset (producing a new analysis for each study removed) and by analysing only data from studies with low risk of bias. Statistical significance was set at the two-tailed 0.05 level for hypothesis testing. Unadjusted p values are reported throughout. This study was performed in compliance with The Cochrane Collaboration and Preferred Reporting Items for Systematic Reviews and Meta-Analyses guidelines [19]–[22] (Checklist S1).

## Results

### Study Characteristics

**Figure 1:**
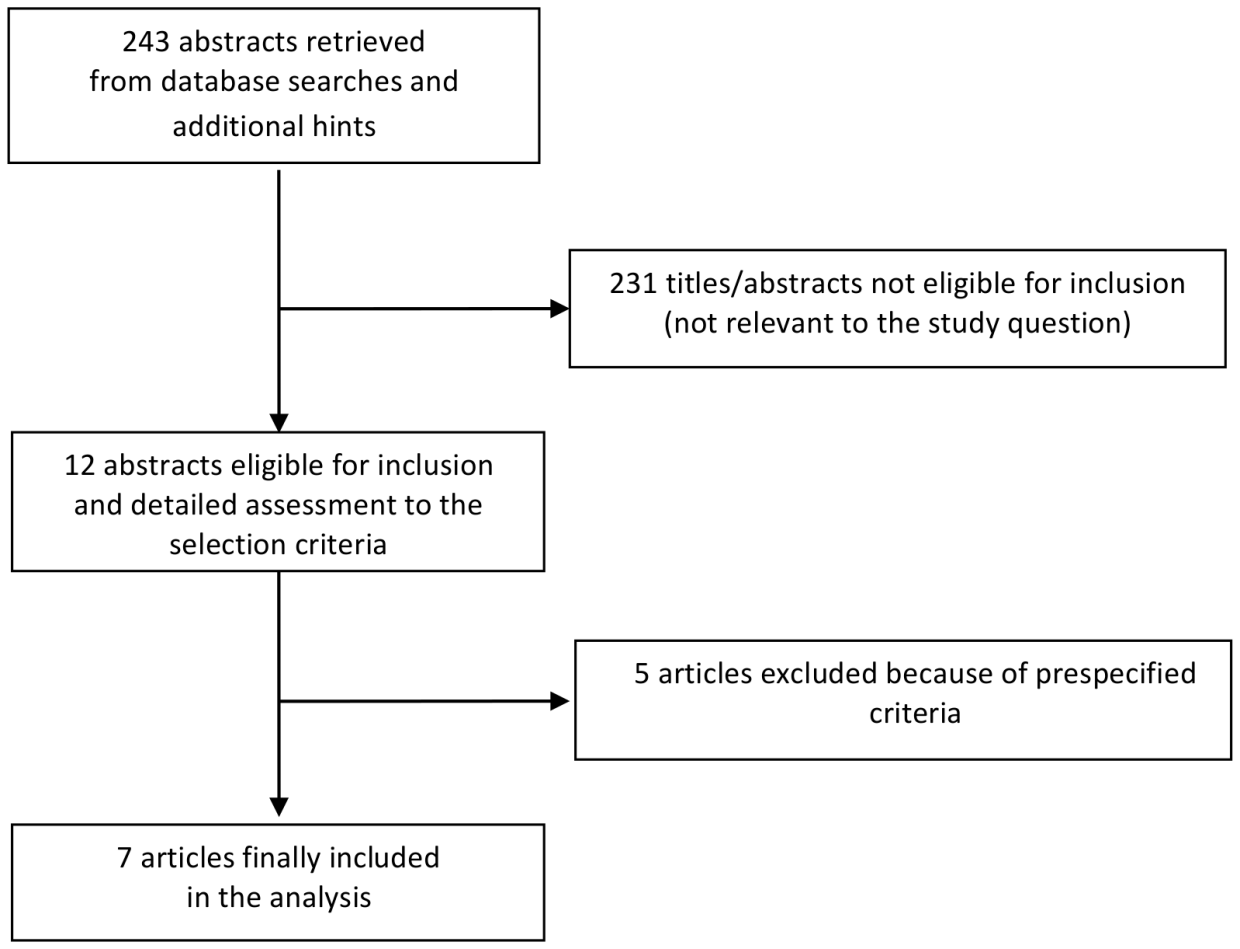
Flow diagram for selection of articles.

Database searches, snowballing, and contacts with experts yielded a total of 257 articles. Excluding 245 non-pertinent titles or abstracts, we retrieved in complete form and assessed 12 studies according to the selection criteria (Figure 1). Seven studies were further excluded because of our prespecified exclusion criteria: three studies were excluded because they were not randomized [23]–[25], three because including not only septic patients [26]–[28] and one because the data were included in a previous publication [29].

The five included manuscripts randomized 650 patients, 322 to statins and 328 to control (Table 1). One of the included trials was multicentre [17]. Clinical heterogeneity was mostly due to setting, statin used, study drug dosage and follow-up duration. Indeed, one trial used statins in severe sepsis [17], three in septic patients in a general ward setting [16], [19], [30] and one in sepsis due to pneumonia [31]. Different statins were used: atorvastatin in four trials [16]–[18], [31] and simvastatin in one trial [30]. Study quality appraisal indicated that four trials were of high quality while one study [31] was published as abstract only (Table 2). The identified comparator was placebo in four trials [16]–[18], [30] while in one trial [31] it was not clearly defined.

**Table 1:** Description of the 5 trials included in the meta-analysis.

First author	Year	Setting	Primary outcome	Statins patients	Controlpatients	Statin used	Statin dose	Comparator	Follow-up	Beneficial effects of statins found	Negative effects of statins found
Choi HS [31]	2008	Sepsis due to pneumonia	ICU and hospital mortality	33	34	Atorvastatin	10 mg daily	Not clearly defined	Hospital discharge	Lower cholesterol level by day 7	None
Kruger P [16]	2011	Sepsis ward	Progression or regression of sepsis during hospital admission	75	75	Atorvastatin	20 mg daily	Placebo	Hospital discharge	None	None
Kruger P [17]	2013	Severe sepsis	Plasma IL-6 levels	123	127	Atorvastatin	20 mg daily	Placebo	90 days	Lower 28-days mortality; lower cholesterol level	None
Novack V [30]	2009	Sepsis ward	Development of severe sepsis	42	41	Simvastatin	40 mg immediatel following enrollment, and once daily 20 mg	Placebo	Hospital discharge	Reduced TNF-a and IL-6 levels	None
Patel JM [18]	2012	Sepsis ward	Progression to severe sepsis	49	51	Atorvastatin	40 mg daily	Placebo	1 year	Significantly lower conversion rate to severe sepsis; lower plasma cholesterol and albumin creatinine ratios at day 4	None

ICU: intensive care unit.

**Table 2:** Methodological quality summary: review authors' judgments about each methodological quality item for each included study.

Domain/question	Adequate sequence generation?	Allocation concealment used?	Blinding?	Concurrent therapies similar?	Incomplete outcome data addressed?	Uniform and explicit outcome definitions?	Free of selective outcome reporting?	Free of other bias?	Overall risk of bias?
Choi HS [31] a	Unclear	Unclear	Yes (single)	Unclear	Unclear	Unclear	Unclear	Unclear	High
Kruger P [16]	Yes	Yes	Yes (double)	Yes	Yes	Yes	Yes	Yes	Very Low
Kruger P [17]	Yes	Yes	Yes (single)	Yes	Yes	Yes	Yes	Yes	Very Low
Novack V[30]	Yes	Yes	Yes (double)	Yes	Yes	Yes	Yes	Yes	Very Low
Patel JM [18]	Yes	Yes	Yes (double)	Yes	Yes	Yes	Yes	Yes	Very Low

^a^ Study published as abstract only.

### Quantitative Data Synthesis

**Figure 2:**
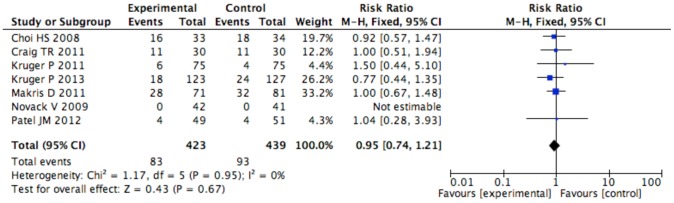
Forest Plot for mortality.

No difference in mortality (Figure 2) was recorded at the longest follow-up available (44/322 [14%] in the statins group vs 50/328 [15%] in the control group, RR = 0.90 [95% CI 0.65 to 1.26], p for effect = 0.6, p for heterogeneity 0.8, I^2^ = 0% with 5 studies included) with results confirmed at sensitivity analyses (Table 3). Switching from random to fixed effects model made no difference to the estimates. Visual inspection of funnel plot did not identify a skewed or asymmetrical shape, excluding the presence of small publication bias (Figure S1).

**Table 3:** Sensitivity analyses of mortality.

Outcome	Number of included trials	Statins patients	Control patients	RR	95% CI	P for effect	P for heterogeneity	I^2^ (%)
**Mortality, events**	5	44/322 [14%]	50/328 [15%]	0.92	0.72 to 1.18	0.6	0.8	0
Monocentric clinical trials [16], [18], [30], [31]	4	26/199 [13%]	26/201[13%]	0.98	0.65 to 1.49	0.7	0.9	0
SENSITIVITY ANALYSIS (including only very low risk of bias studies)	4	28/289[10%]	32/294[11%]	0.90	0.55 to 1.43	0.6	0.6	0
SENSITIVITY ANALYSIS (removing 1 study at time)	All 95% CIs of RR<1 and p>0.05							

RR: Risk Ratio; CI: confidence interval; P: statistical p-value; I^2^: I square.

Each single study showed improvements in secondary endpoints such as organ dysfunction, ventilator associated pneumonia or inflammatory markers (Table 1) but this did not translate in difference in hospital stay (SMD = −0.24 [−1.59 to 1.12] days, p for effect = 0.7, p for heterogeneity 0.20, I^2^ = 36% with 4 studies and 583 patients included). Visual inspection of funnel plot did not identify a skewed or asymmetrical shape, excluding the presence of small publication bias (Figure S2).

## Discussion

Our meta-analysis shows that statins therapy does not influence mortality in septic patients. This is the first meta-analysis ever performed on this topic that includes only randomized clinical trials.

In recent years the use of statins in critically ill patients has particularly attracted intensive care physicians but publications had discordant results probably because of the high heterogeneity of the included studies and because of the poor quality of non-randomized trials.

The growing interest in the use of statins in sepsis is derived from some experimental and subsequent clinical studies demonstrating a beneficial effect of statins during acute respiratory distress syndrome, acute lung injury or sepsis. In fact two experimental animal studies showed an improved survival in animals treated with statins before induction of sepsis [32], [33]. The results of many subsequent clinical studies were summarized in an interesting meta-analysis supporting the hypothesis of a protective effect of statins during sepsis [34]. This previous systematic review included 20 clinical trials, all but one [26] observational, 15 of which showing a decreased mortality rate in patients receiving statins.

Chopra et al. [35], in a recent meta-analysis on the effects of statins on mortality of patients with community-acquired pneumonia, showed that statin use was associated with an improved 30-day survival, but this beneficial effect weakened in important subgroups of patients and in high-quality methodological studies.

Trying to understand the actual role of statin therapy in critically ill patients is mandatory. While it's true that statins are probably the most prescribed drugs in the world [36] and a potential aid in reducing mortality in septic patients would be desirable, the impact of their possible side effects in critically ill patients should not be underestimated.

It is well known that sepsis is characterized by systemic inflammation and impairment of the coagulation cascade [37] and the pleiotropic effect of statins in this setting may, therefore, be beneficial. Instead, what is not yet well known, is the incidence and severity of statins side effects in critically ill patients. Their most severe side effects, myopathy and rhabdomyolysis, are really rare in the generally population [38], but it can't be excluded that their incidence and severity could be higher in compromised patients, with a theoretical consequent detrimental effect on survival.

Our study found no evidence of a beneficial effect of statins therapy on mortality in septic patients. The strength of our analysis is that it includes only randomized clinical trials, the preferred study design to assess the efficacy of a medical treatment. On the other hand, however, the few included studies and the small number of patients in these RCTs, don't allow to draw definitive conclusions on the real role of statins therapy in critically ill patients.

### Limitations

We acknowledge that this study has several limitations. First of all it includes a limited number of small clinical trials, all but one monocentric. Moreover studies present clinical heterogeneity (setting, statin used, study drug dosage and follow-up duration). Despite the pooled sample size (650 patients in five RCTs), we cannot conclude whether the lack of a statistically significant improvement in survival was due to inadequate power or due to a true lack of beneficial effects of statins. In fact, with 650 patients and a mortality rate of 15% in the control group, statins had to reduce mortality by an implausible 50% (from 15% to 7.5%) to obtain a statistically significant result. Nonetheless, the results of our meta-analysis are useful for future researchers in that, assuming a 15% mortality in the control group, a plausible 25% reduction in mortality in the statins group (from 15% to 11.25%) and a power of 80% you have to randomize 1325 patient per group to have an adequately powered RCT. Moreover, given the small number of studies, we were unable to evaluate the role of statins in specific subsettings or on other relevant clinical outcomes such as length of intensive care unit stay or length of mechanical ventilation.

### Conclusions

Even if all randomized data published so far show that statins therapy has beneficial effect on secondary outcomes or inflammatory markers, this meta-analysis of randomized trials showed no effect of statins on mortality or length of hospital stay in the overall population of adult septic patients. Scientific evidence on statin role in septic patients is still limited and larger randomized clinical trials should be performed on this topic.

## Supporting Information

Text S1 **Full PubMed search strategy.**
(DOCX)

Figure S1 **Funnel Plot for mortality.**
(TIF)

Figure S2 **Funnel Plot for HLOS.**
(TIF)

Checklist S1 **PRISMA checklist.**
(DOC)
