# Supplementary material for: The Effect of Statins on Mortality in Septic Patients: A Meta-Analysis of Randomized Controlled Trials
Source: PLoS One. 2013 Dec 31;8(12):e82775. doi: 10.1371/journal.pone.0082775 (PMC3876996; doi:10.1371/journal.pone.0082775)
Supplement: Text S1 — Full PubMed search strategy. (DOCX) [file pone.0082775.s001.docx]

**Text S1: Full PubMed search strategy**

(((statin*[tiab] OR ((“hydroxymethylglutaryl-CoA reductase”[tiab] OR "HMGCoA reductase"[tiab]) AND inhibitor*[tiab]) OR anticholesteremic[tiab] OR simvastatin[tiab] OR rosuvastatin*[tiab] OR pravastatin*[tiab] OR atorvastatin*[tiab] OR fluvastatin*[tiab] OR cerivastatin*[tiab] OR pitavastatin*[tiab] OR lovastatin*[tiab]) AND (sepsis[tw] OR infection[mh] OR pneumonia[mh] OR "acute lung injury" OR "Respiratory Distress Syndrome” OR "Critical Illness”[mh])) AND (randomized controlled trial[pt] OR controlled clinical trial[pt] OR randomized controlled trials[mh] OR random allocation[mh] OR double-blind method[mh] OR single-blind method[mh] OR clinical trial[pt] OR clinical trials[mh] OR (clinical trial[tw] OR ((singl*[tw] OR doubl*[tw] OR trebl*[tw] OR tripl*[tw]) AND (mask*[tw] OR blind[tw])) OR (latin square[tw]) OR placebos[mh] OR placebo*[tw] OR random*[tw] OR research design[mh:noexp] OR follow-up studies[mh] OR prospective studies[mh] OR cross-over studies[mh] OR control*[tw] OR prospectiv*[tw] OR volunteer*[tw]))) NOT ((animal[mh] NOT human[mh]))
