# Supplementary figures and images for: The Effect of Statins on Mortality in Septic Patients: A Meta-Analysis of Randomized Controlled Trials
Source: PLoS One. 2013 Dec 31;8(12):e82775. doi: 10.1371/journal.pone.0082775 (PMC3876996; doi:10.1371/journal.pone.0082775)

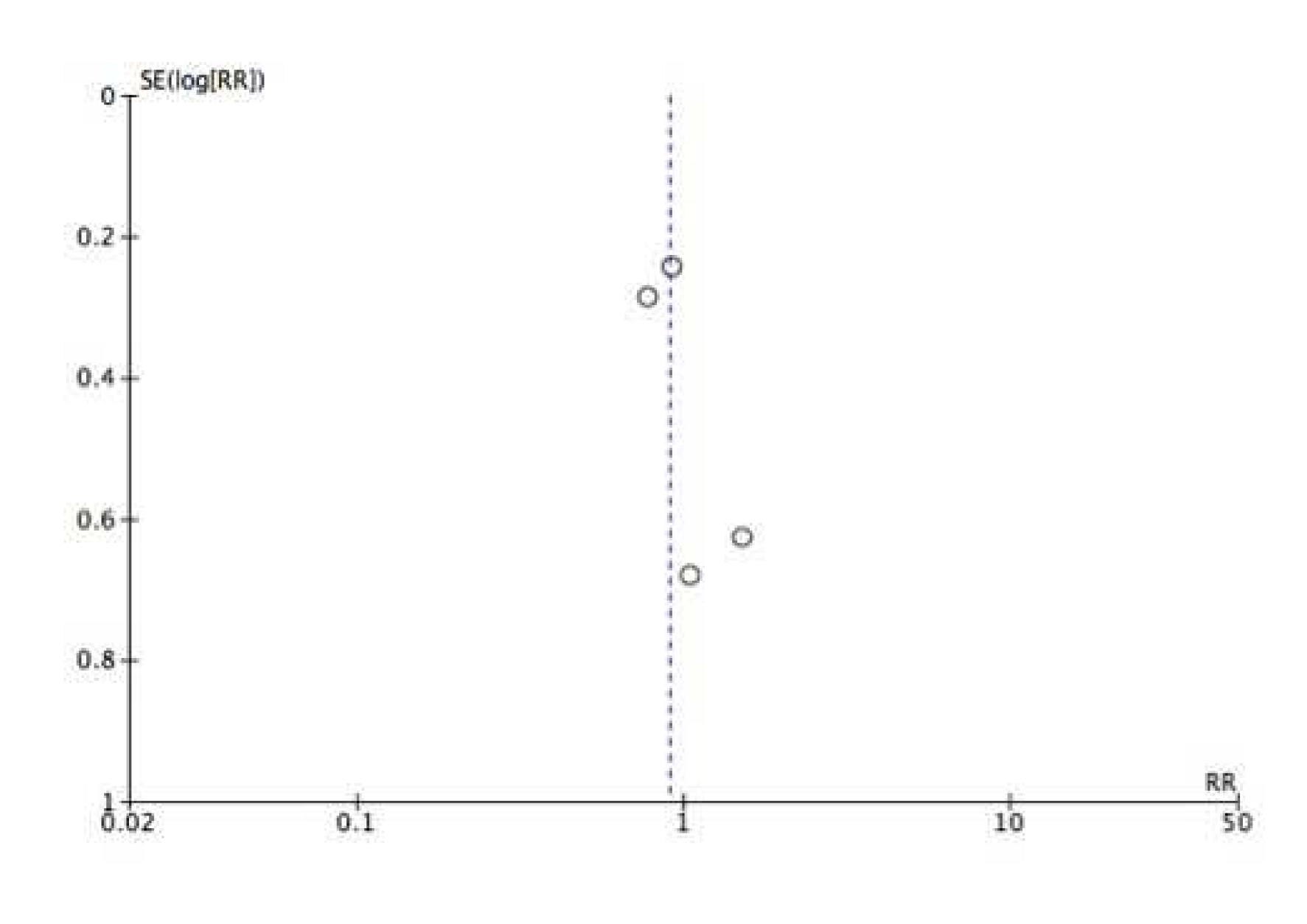

Supplement: Figure S1 — Funnel Plot for mortality. (TIF) [file pone.0082775.s002.tif]

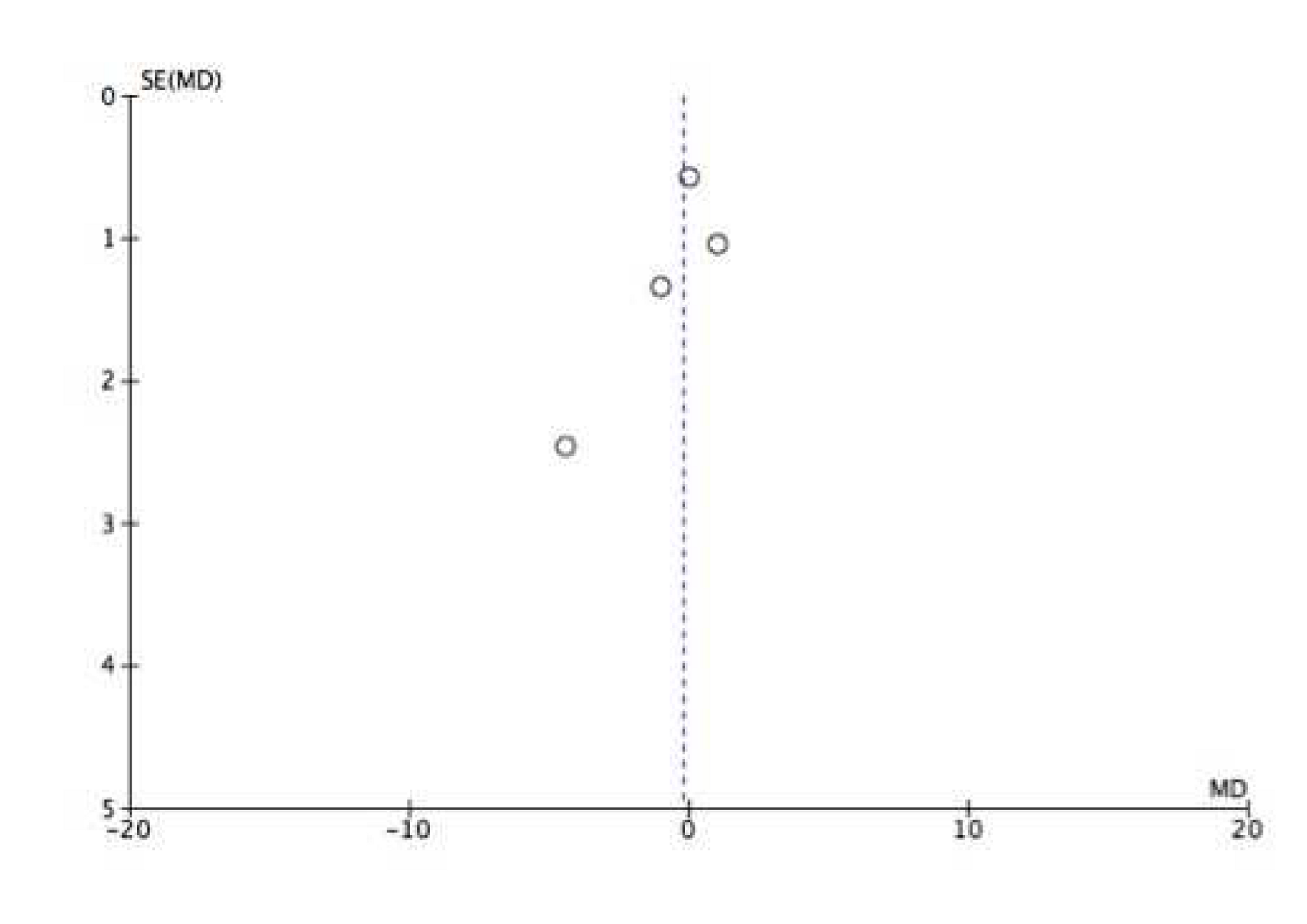

Supplement: Figure S2 — Funnel Plot for HLOS. (TIF) [file pone.0082775.s003.tif]
